# Effects of Multi-Species Direct-Fed Microbial Products on Ruminal Metatranscriptome and Carboxyl-Metabolome of Beef Steers

**DOI:** 10.3390/ani11010072

**Published:** 2021-01-02

**Authors:** Megan McCoun, Adeoye Oyebade, Zaira M. Estrada-Reyes, Andres A. Pech-Cervantes, Ibukun M. Ogunade

**Affiliations:** 1 Division of Food and Animal Science, Kentucky State University, Frankfort, KY 40601, USA; xtiemira@gmail.com; 2 Department of Animal Sciences, University of Florida, Gainesville, FL 32611, USA; adeoye.oyebade@ufl.edu; 3 College of Agricultural, Family Sciences, and Technology, Fort Valley State University, Fort Valley, GA 31030, USA; zaira.estradareyes@fvsu.edu (Z.M.E.-R.); andres.pechcervantes@fvsu.edu (A.A.P.-C.); 4 Division of Animal and Nutritional Science, West Virginia University, Morgantown, WV 26506, USA

**Keywords:** fatty acid peroxidation, metabolome, lactic acid bacteria, rumen fluid, *Saccharomyces cerevisiae*

## Abstract

**Simple Summary:**

This study examined the effects of two direct-fed microbial (DFM) products containing multiple microbial species and their fermentation products on ruminal metatranscriptome and carboxyl-metabolome of beef steers. The two DFM products altered the relative concentrations of short-chain fatty acids. No effects were detected on the functional attributes of the rumen microbiota; however, one of the two DFM products reduced the relative concentrations of metabolites involved in fatty acid peroxidation and amino acid degradation. This study demonstrated that dietary supplementation with either PROB or SYNB altered the ruminal fermentation pattern. In addition, supplemental PROB reduced the relative concentrations of metabolic products of fatty acid peroxidation and amino acid degradation.

**Abstract:**

We examined the effects of two direct-fed microbial (DFM) products containing multiple microbial species and their fermentation products on ruminal metatranscriptome and carboxyl-metabolome of beef steers. Nine ruminally-cannulated Holstein steers were assigned to 3 treatments arranged in a 3 × 3 Latin square design with three 21-d periods. Dietary treatments were (1) Control (CON; basal diet without additive), (2) Commence (PROB; basal diet plus 19 g/d of Commence), and (3) RX3 (SYNB; basal diet plus 28 g/d of RX3). Commence and RX3 are both *S. cerevisiae*-based DFM products containing several microbial species and their fermentation products. Mixed ruminal contents collected multiple times after feeding on day 21 were used for metatranscriptome and carboxyl-metabolome analysis. Partial least squares discriminant analysis revealed a distinct transcriptionally active taxonomy profiles between CON and each of the PROB and SYNB samples. Compared to CON, the steers fed supplemental PROB had 3 differential (LDA ≥ 2.0; *p* ≤ 0.05) transcriptionally active taxa, none of which were at the species level, and those fed SYNB had eight differential (LDA > 2.0, *p* ≤ 0.05) transcriptionally active taxa, but there was no difference (*p* > 0.05) between PROB and SYNB. No functional microbial genes were differentially expressed among the treatments. Compared with CON, 3 metabolites (hydroxylpropionic acid and 2 isomers of propionic acid) were increased (FC ≥ 1.2, FDR ≤ 0.05), whereas 15 metabolites, including succinic acid and fatty acid peroxidation and amino acid degradation products were reduced (FC ≤ 0.83, FDR ≤ 0.05) by supplemental PROB. Compared with CON, 2 metabolites (2 isomers of propionic acid) were increased (FC ≥ 1.2, FDR ≤ 0.05), whereas 2 metabolites (succinic acid and pimelate) were reduced (FC ≤ 0.83, FDR ≤ 0.05) by supplemental SYNB. Compared to SYNB, supplemental PROB reduced (FC ≤ 0.83, FDR ≤ 0.05) the relative abundance of four fatty acid peroxidation products in the rumen. This study demonstrated that dietary supplementation with either PROB or SYNB altered the ruminal fermentation pattern. In addition, supplemental PROB reduced concentrations of metabolic products of fatty acid peroxidation and amino acid degradation. Future studies are needed to evaluate the significance of these alterations to ruminal fatty acid and amino acid metabolisms, and their influence on beef cattle performance.

## 1. Introduction

Direct-fed microbials (DFM) are fed as a source of active and naturally occurring microorganisms in livestock production systems to improve the health and productivity of animals [1]. The use of DFM in livestock diet is a popular practice due to public concern over the use of antibiotics in livestock production [1]. *Saccharomyces cerevisiae*, lactic acid-utilizing and lactic acid-producing bacteria are the most commonly used DFM in ruminant production system [2]. In recent years, most *S. cerevisiae*-based microbial additives are formulated to contain a combination of several microbial species or their fermentation products, in order to ensure efficacies and multi-factorial response to their supplementation [2].

Several studies utilized culture-independent molecular techniques to study the modes of action of DFM products, which include shift in rumen microbial community and fermentation pattern [3,4]. Most of the studies that evaluated a microbial community shift focused primarily on relative microbial abundance in the rumen [4,5]. However, how DFM affects the functional activity (analyzed via metatranscriptomics) of the rumen microbiome is currently unknown. Furthermore, the effects of microbial additives on an altered ruminal fermentation pattern caused by rumen microbiome shift primarily focused on ruminal short chain fatty acids, such as volatile fatty acid profile. However, little or no emphasis was placed on how DFM affects the metabolism of other carboxylic acid-containing metabolites (carboxyl-metabolome), such as fatty acid metabolites in the rumen.

Chemical isotope labeling liquid chromatography mass spectrometry (LC-MS)-based metabolomics provided an opportunity to quantitatively analyze several carboxylic acid-containing metabolites in the biofluids [6]. Global profiling of carboxylic acid-containing metabolites in the rumen is needed, because a vast proportion of the metabolites, such as fatty acid metabolites and amino acids, in the rumen, contain at least one carboxylic acid group in their chemical structure. We had previously demonstrated that dietary supplementation of either of two multi-species DFM products improved the energy status and altered the ruminal bacterial community of beef steers [7]; however, taxonomically dissimilar microbiomes can share similar metabolic functions. Therefore, determining the effects of these DFM products on the functional attributes of the rumen microbiome and carboxyl-metabolome would enhance our understanding of their mechanisms of action. Thus, this study investigated the effects of two multi-species DFM products on ruminal metatranscriptome and carboxyl-metabolome of beef steers. 

## 2. Materials and Methods

Nine ruminally cannulated Angus beef steers with a mean body weight (BW) of 243 ± 12 kg were assigned to 3 treatments in a 3 × 3 Latin square design, with three 21-d periods and 10-d wash-out between periods. The steers were individually fed (3% BW) a total mixed ration containing 20.3% concentrate mix and 79.7% corn silage on a dry matter basis (Table 1), once daily at 09:00 am. Dietary treatments were (1) Control (CON; basal diet without additive), (2) Commence (PROB; basal diet plus 19 g/d of Commence), and (3) RX3 (SYNB; basal diet plus 28 g/d of RX3). Commence is an optimized blend of live *S. cerevisiae*, *Enterococcus lactis*, *Bacillus subtilis*, *Enterococcus faecium*, *and L. case*i, and their fermentation products. RX3 is an optimized blend of active *S. cerevisiae* and the fermentation products of *S. cerevisiae, Enterococcus lactis*, *Bacillus licheniformis*, and *Bacillus subtilis*. The DFM products were top-dressed daily on the total mixed ration, in the form of a premix using dried distillers grain with solubles, while a similar premix with no additive was top-dressed for the CON treatment.

### 2.1. Rumen Fluid Sampling

On day 21 of each period, 150 mL of the ruminal contents were collected via the cannula at 1, 3, 6, 9, 12, and 18 h after feeding. A sample of the rumen content was manually homogenized, as described by previous studies [4,8]. In brief, the ruminal contents were hand-strained through 4 layers of cheesecloth to separate the solid and liquid fractions. The samples of the solid and liquid phases for each collection time were composited for each steer and mixed 1:1 (w/w). Due to the dynamic nature of the rumen microbiota and fermentation, mixed rumen fluid samples collected multiple times within a day is needed to reduce or eliminate variation due to the time of collection. Approximately 150 mL of the mixed rumen contents was then mixed with 100 mL of RNAlater (Thermo Fisher Scientific, Waltham, MA, USA) to prevent RNA degradation, and was stored at −80 °C, until RNA extraction and sequencing were done. Another sub-sample of the mixed rumen content was strained through 4 layers of cheesecloth to separate the solid and liquid fractions, and the liquid fraction was composited for each steer and was immediately stored at −80 °C, until the metabolomics analysis.

### 2.2. Metatranscriptomics Analysis

Approximately 0.25 g of each ruminal sample underwent RNA extraction using the RNeasy PowerMicrobiome Kit (Qiagen, Valencia, CA, USA), following the manufacturer’s protocol. The RNeasy PowerMicrobiome kit utilizes a cell lysis protocol, which is based on the addition of each sample to a beaded tube, in combination with Lysis buffer (Solution PM1/β- mercaptoethanol). All RNA extracts were quantified using the Qubit RNA High Sensitivity Kit (Invitrogen, Carlsbad, CA, USA), to confirm complete DNase treatment of the RNA extracts (DNA concentration < 0.05 ng/μL). Subsequently, approximately 100 ng of extracted RNA was subjected to NEBNext Ultra RNA Library Prep (New England BioLabs, Ipswich, MA, USA) double-stranded cDNA synthesis and metatranscriptome library preparation. Quality of the final library was assessed using a high sensitivity bio-analyzer chip (Agilent, Santa Clara, CA, USA). Purified metatranscriptome libraries underwent sequencing on the Illumina HiSeq4000, following a 2 × 150 bp index run at the UC Davis Genome Center. Raw read quality was assessed using the program FastQC to obtain average Q scores across the read length of all R1 and R2 sequences. The program fastp was utilized to quality-filter the raw sequence data. A sliding window filtration was utilized to cut reads at a 4-base average Q score of 28 or lower, reads trimmed below 90 basepairs were discarded [9]. After filtration, the reads were subjected to host, *Bos taurus*, cDNA subtraction using Kraken2 [10].

### 2.3. CIL-LC/MS-Based Metabolomics Analysis

Relative quantification of metabolites containing the carboxylic acid chemical group (carboxyl-metabolome) in the rumen fluid samples was done using an CIL/LC-MS-based metabolomics technique [11]. The CIL/LC-MS technique applied the use of isotope-coded p-dimethylaminophenacyl bromide as a reagent to label carboxylic acid-containing metabolites. One (1) mL subsample of the rumen liquid sample was centrifuged at 15,000× *g* for 10 min and the supernatant was separated for further analysis. Normalization of the sample amount was done using liquid chromatography–ultraviolet, as previously described [12]. Relative quantification of the metabolites based on peak ratio values was performed on an LC system (Palo Alto, CA, USA) connected to a Bruker Impact HD quadrupole time-of-flight MS (Billercia, MA, USA). Detailed information of the CIL-LC/MS-based metabolomics protocol was previously reported [11,13]. A quality control sample, prepared by mixing equal amount of a ^12^C-labeled and a ^13^C-labeled pooled sample was injected every 9 runs, to assess the performance of the instrument.

### 2.4. Data and Statistical Analysis

#### 2.4.1. Metatranscriptomics Analysis

Taxonomy annotation was done using the Kraken2 software package, with an elevated confidence score threshold of 0.1, on the filtered reads [10]. Alpha diversity rarefaction curves were created within the QIIME 1.9.1, package using an unnormalized bacterial count table [14]. Multiple rarefactions were performed on all samples using a minimum depth of 2,000,000 sequences to a maximum depth of 20,000,000 sequences, with a step size of 2,000,000 sequences for 10 iterations. Rarefactions were then collated and compared between CON and each of PROB and SYNB, based on the Chao1 metrics. Alpha diversity comparison was conducted using a two-sample *t*-test and non-parametric Monte Carlo permutations (N = 999) within QIIME-1.9.1. Partial least squares discriminant analysis (PLS-DA) was performed using cumulative-sum scaling normalized bacterial abundance data, using the mixOmics R package [15]. The PLS-DA model was trained using a 10-fold cross validation, and this model underwent 150 iterations. Counts per million (CPM) normalized abundances of the taxonomic profiles were formatted, as described in [16]. Linear discriminant analysis Effect Size (Lefse) comparisons were made between CON and each of PROB and SYNB groups. Alpha levels of 0.05 were used for both the Kruskal–Wallis and pairwise Wilcoxon tests. LDA scores greater than 2.0 are displayed for taxonomy. The resulting taxonomic biomarkers between the CON and PROB samples were identified and then plotted as a differential feature abundance plots. Functional gene annotation and quantification of filtered sequence data were conducted using HUMAnN2 [17]. Filtered reads were first mapped against the Uniref90 functional gene database, which were subsequently regrouped as eggNOG orthologies. Reads per kilobase counts underwent CPM normalization to account for differences in sequencing depth among samples, resulting in a final read per kilobase per million data matrix. Linear discriminant analysis effect size comparisons were used to identify differentially expressed microbial functional genes among the treatments. Alpha levels of 0.05 were used for both the Kruskal–Wallis and pairwise Wilcoxon tests. Functional genes with LDA > 2.0 and *p*-value ≤ 0.05 were considered to be differentially expressed.

#### 2.4.2. Metabolomics Data Analysis

The metabolomics data were analyzed using Bruker Data Analysis 4.4 (Mung and Li, 2017). The peak pairs that were present in at least 80% of samples in any treatment group were retained. The data were normalized by ratio of total useful signal. Peak pairs were identified, based on accurate mass and retention or predicted retention time matches using a labeled metabolite library (CIL Library; [18]) and a linked identity library (LI Library; [19]). All other peak pairs were unidentified. Partial least squares discriminant analysis (PLS-DA) scores plot was used to visualize the differences among the treatments. Volcano plot was constructed by plotting the fold change of each metabolite against the FDR (false discovery rate) adjusted *p-value*. The fold change was calculated as Mean [20]/Mean [21], Mean(SYNB)/Mean [21], and Mean [20]/Mean(SYNB). The criterion used was FC ≥ 1.2 or ≤0.83 and FDR adjusted *p-value* ≤ 0.05.

## 3. Results

The effects of PROB and SYNB supplementation on ruminal bacterial community analyzed via 16S rRNA sequencing, volatile fatty acid and ammonia-*N* concentrations, and energy status of the beef steers were previously reported in our companion paper [7].

### 3.1. Effects of PROB and SYNB on Ruminal Metatranscriptome Profile

**Figure 1:**
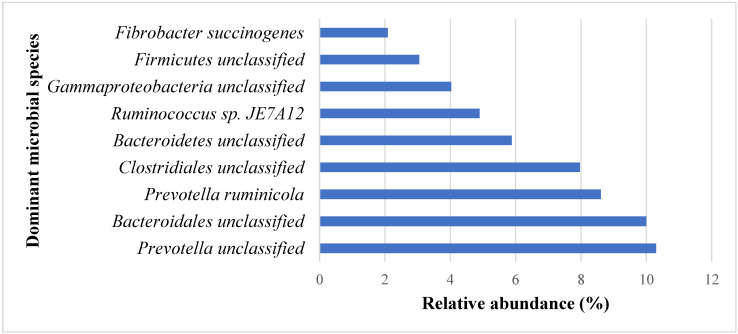
Relative abundance (%) of the 9 most dominant transcriptionally active taxa at the species level.

Sequencing results revealed that a range of 20–82 million clusters for all samples were retained after quality filtration and read merging (average of 38.3 million clusters per sample). The nine (9) most abundant transcriptionally active taxa at the species level, which constitute 56.8% of the total microbial community, are shown in Figure 1. The relative abundance of all transcriptionally active taxa detected in all samples is shown in Supplementary Table S1. Unclassified species belonging to *Prevotella* (10.3 ± 4.0) and *Bacteroidales* (10.0 ± 2.9 %), and *Prevotella ruminicola* (8.6 ± 4.9 %) were the most abundant transcriptionally active taxa at the species level (Figure 1).

**Figure 2:**
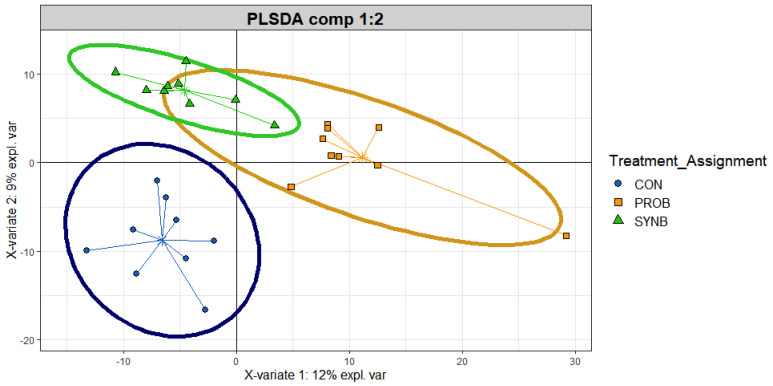
Partial least squares discriminant analysis scores plot of the ruminal transcriptionally active microbial community of beef steers fed diet, supplemented with direct-fed microbials containing multiple microbial species and their fermentation products. CON = control; PROB = a blend of *S. cerevisiae*, *Enterococcus lactis, Bacillus subtilis, Enterococcus faecium, and L. case*i, and their fermentation products fed at 19 g/steer/day (PMI, Arden Hills, MN); SYNB = a blend of live *S. cerevisiae* and the fermentation products of *S. cerevisiae, Enterococcus lactis*, *Bacillus licheniformis*, and *Bacillus subtilis* fed at 28 g/steer/day PMI, Arden Hills, MN).

**Figure 3:**
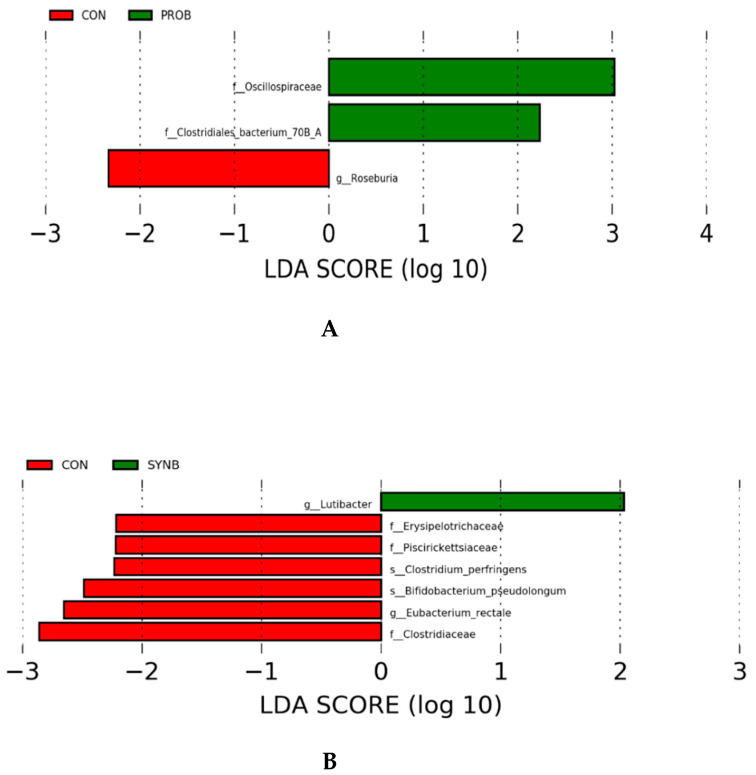
Linear discriminant analysis effect size of the ruminal transcriptionally active microbial community of beef steers fed diet supplemented with direct-fed microbials containing multiple microbial species and their fermentation products. (**A**) CON vs. PROB, (**B**) CON vs. SYNB. CON = control; PROB = a blend of *S. cerevisiae*, *Enterococcus lactis, Bacillus subtilis, Enterococcus faecium, and L. case*i, and their fermentation products fed at 19 g/steer/day (PMI, Arden Hills, MN); SYNB = a blend of live *S. cerevisiae* and the fermentation products of *S. cerevisiae, Enterococcus lactis*, *Bacillus licheniformis*, and *Bacillus subtilis* fed at 28 g/steer/day PMI, Arden Hills, MN).

Partial least squares discriminant analysis revealed a distinct transcriptionally active taxonomic profiles between CON and each of the PROB and SYNB samples, but there was an overlap between the PROB and SYNB (Figure 2). Linear discriminant analysis effect size analysis of the taxonomic profile revealed no differential transcriptionally active taxa between the PROB and SYNB samples (data not shown). A total of 3 differential (LDA > 2.0, *p* ≤ 0.05) transcriptionally active taxa were detected between the CON and PROB samples, none of which were at the species level (Figure 3A). Eight differential (LDA > 2.0, *p* ≤ 0.05) transcriptionally active taxa were detected between the CON and SYNB samples (Figure 3B), two of which (*Clostridium perfringens* and *Bifidobacterium pseudolongum*) were the only differential transcriptionally active taxa at the species level and were enriched in CON compared to SYNB. Results of the functional gene annotation revealed no microbial genes were differentially expressed among the treatments (LDA < 1; Supplementary Figure S1).

### 3.2. Effects of PROB and SYNB on Ruminal Carboxyl-Metabolome

**Figure 4:**
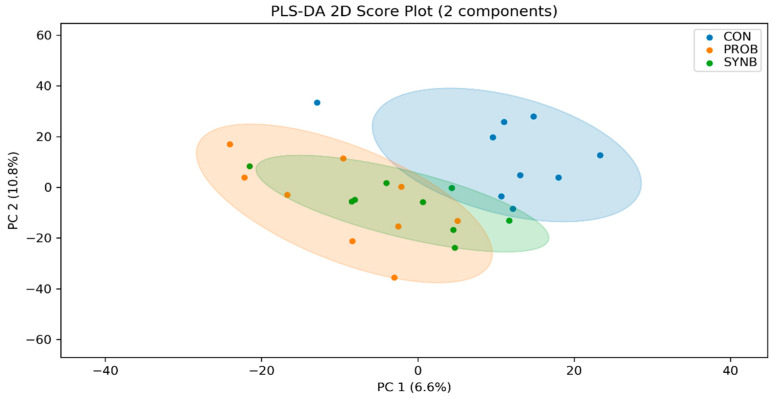
Partial least squares discriminant analysis (PLS-DA) scores plots of the ruminal metabolome of the beef steers fed diets supplemented with direct-fed microbials containing multiple microbial species and their fermentation products. CON = control; PROB = a blend of *S. cerevisiae*, *Enterococcus lactis, Bacillus subtilis, Enterococcus faecium, and L. case*i, and their fermentation products fed at 19 g/steer/day (PMI, Arden Hills, MN); SYNB = a blend of live *S. cerevisiae* and the fermentation products of *S. cerevisiae, Enterococcus lactis*, *Bacillus licheniformis*, and *Bacillus subtilis* fed at 28 g/steer/day PMI, Arden Hills, MN).

An average of 2386 ± 54 peak pairs per sample were detected and a total number of 221 peak pairs were identified (Supplementary Table S2). Partial least squares discriminant analysis (PLS-DA) scores plot revealed separations between the metabolome of CON and each of the PROB and SYNB groups (Figure 4).

**Figure 5:**
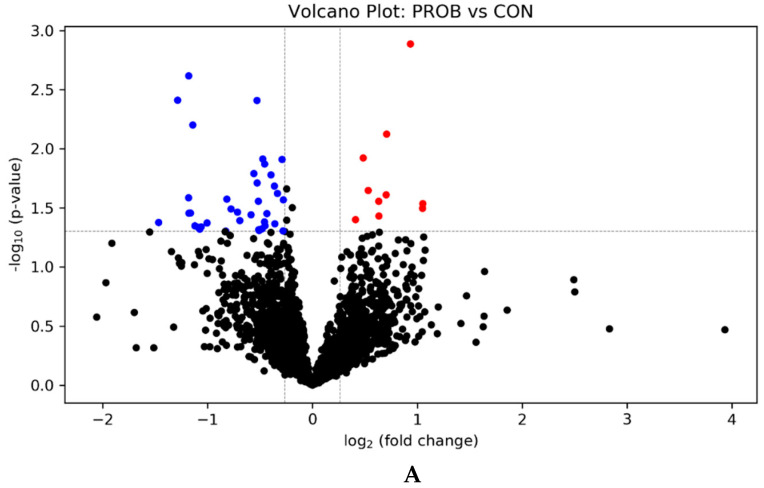

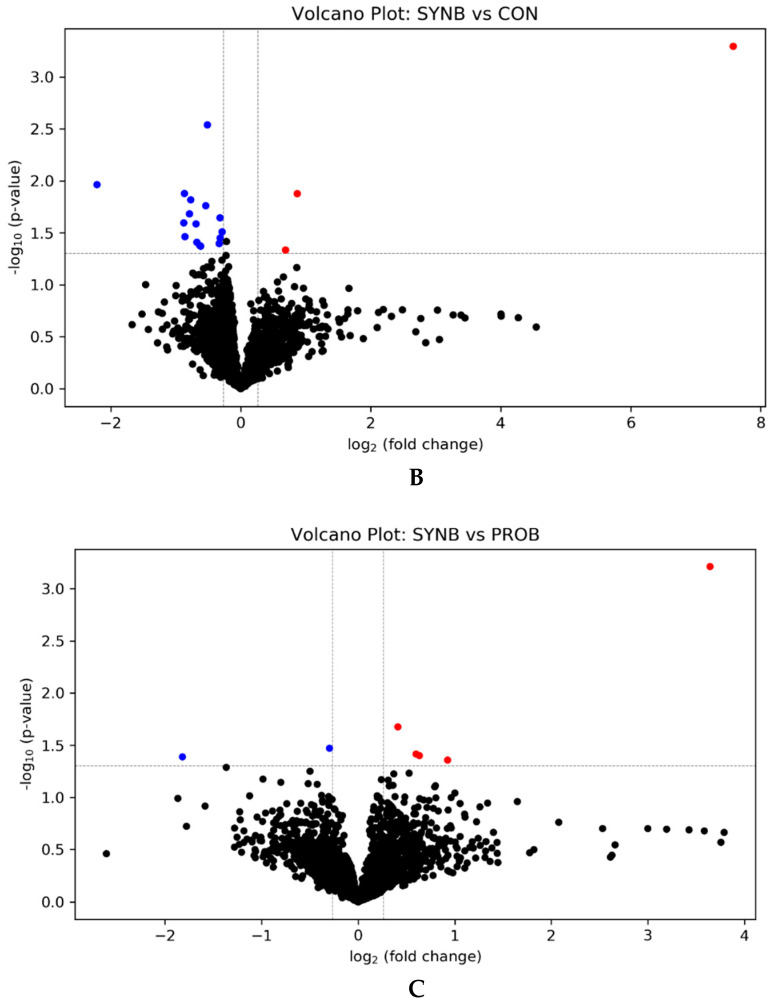
Volcano plots (**A**) CON vs. PROB, (**B**) CON vs. SYNB, and (**C**) PROB vs. SYNB, showing the differential ruminal metabolites in beef steers fed diets supplemented with *S. cerevisiae*-based direct-fed microbial products. FC ≥ 1.2, *p-value* ≤ 0.05 (in red)—significantly increased; FC ≤ 0.83, *p-value* ≤ 0.05 (in blue)—significantly reduced relative to CON. CON = control; PROB = a blend of *S. cerevisiae*, *Enterococcus lactis, Bacillus subtilis, Enterococcus faecium, and L. case*i, and their fermentation products fed at 19 g/steer/day (PMI, Arden Hills, MN); SYNB = a blend of live *S. cerevisiae* and the fermentation products of *S. cerevisiae, Enterococcus lactis*, *Bacillus licheniformis*, and *Bacillus subtilis* fed at 28 g/steer/day (PMI, Arden Hills, MN).

A total of 47 differential (FDR ≤ 0.05) peak pairs (18 of which were identified as metabolites) were detected between the CON and PROB (Figure 5A); three metabolites (hydroxylpropionic acid and 2 isomers of propionic acid) were increased (FC ≥ 1.2, FDR ≤ 0.05), whereas 15 metabolites (succinic acid, 7 isomers of 9-oxononanoic acid, 3 isomers of 12,13-epoxy-9-hydroxy-10-octadecenoate, 2-amino-5-oxohexanoate, 6-acetamido-2-oxohexanoate, 8-iso prostaglandin F1alpha, and 9,10-epoxy-13-hydroxy-11-octadecenoate) were reduced (FC ≤ 0.83, FDR ≤ 0.05) by supplemental PROB (Table 2).

A total of 19 differential (FDR ≤ 0.05) peak pairs (4 of which were identified as metabolites) were detected between the CON and the SYNB groups (Figure 5B); 2 metabolites (2 isomers of propionic acid) were increased (FC ≥ 1.2, FDR ≤ 0.05) whereas 2 metabolites (succinic acid and pimelate) were reduced (FC ≤ 0.83, FDR ≤ 0.05) by supplemental SYNB (Table 2). When PROB and SYNB were compared, a total of 7 differential (FDR ≤ 0.05) peak pairs (4 of which were identified as metabolites) were detected between the PROB and the SYNB groups (Figure 5C); the 4 metabolites (2 isomers of 9-oxononanoic acid, 12,13-epoxy-9-hydroxy-10-octadecenoate, and 9,10-epoxy-13-hydroxy-11-octadecenoate) were reduced (FC ≤ 0.83, FDR ≤ 0.05) by supplemental PROB, relative to SYNB (data not shown).

## 4. Discussion

*Prevotella* spp. are considered the most dominant bacterial group in the rumen and were reported to grow rapidly in the presence of fermentable carbohydrates, such as those contained in corn silage [22,23,24]. Similar to this study, the *Prevotella* species were the most dominant metabolically active species in the rumen of dairy cows fed 100% corn silage-based diet [25]. Compared with CON, dietary supplementation of SYNB reduced the relative abundance of *Clostridium perfringens* and *Bifidobacterium pseudolongum. Clostridium perfringens* is a bacterial pathogen that causes intestinal disease in both animals and humans [26], while the role of *Bifidobacterium pseudolongum* in the rumen is not adequately described. This result is consistent with previous studies that reported the pathogen exclusion effects of DFM products against *Clostridium perfringens*, *E. coli*, and *Salmonella* [27,28] via several mechanisms, including direct binding to the intestinal epithelium, production of compounds that are toxic to the pathogens, or competition for nutrients [2,29].

No differentially expressed microbial functional genes were detected among the treatments, indicating that the functional activity of the ruminal microbiota was not altered by the dietary supplementation of PROB or SYNB. This was in line with little or no effects on the ruminal active microbial taxonomy observed in this study. To the best of our knowledge, this was the first study that attempted to investigate the effects of *S. cerevisiae*-based DFM on the relative abundance of transcriptionally active members and functional attributes of the rumen microbiota in beef cattle that were fed a corn silage-based diet. Several previous studies that evaluated the effects of *S. cerevisiae*-based DFM on rumen microbial population, focused on the taxonomic abundance of rumen microbial population [4,30,31]. In our companion study [7], we analyzed the effects of supplemental PROB and SYNB on the relative abundance of ruminal bacterial via 16S rRNA gene sequencing and observed that, compared to CON, supplemental PROB altered the relative abundance of *Succinivibrio*, *Rikenellaceae* RC9 gut group, *Succiniclasticum*, *Prevotella* 1, and *Prevotellaceae* UCG-001, whereas SYNB altered the relative abundance of *Succinivibrionaceae UCG-*001, *Succiniclasticum*, *Prevotella* 1, and *Prevotellaceae* UCG-001. However, several studies demonstrated that taxonomically dissimilar microbiomes can share similar metabolic functions [21,32]. Indeed, animal phenotypes were reported to be more closely associated with rumen microbial functional profiles than taxonomic profiles [21,33]. It is important to note that we might not have captured the lowly expressed microbial gene, due to the sequencing depth of the metatranscriptomic dataset used in this study (average of 38.3 million clusters per sample). However, to date, the appropriate sequencing depth that is sufficient to capture all microbial genes in the rumen is not yet known [32].

Alteration in the concentrations of volatile fatty acids, such as increased concentration of propionic acid (and its isomers) and decreased concentration of succinic acid caused by supplemental PROB or SYNB is an indication of altered rumen fermentation pattern. Several studies demonstrated that additives containing *S. cerevisiae* altered the rumen fermentation pattern towards increased proportion of propionate, the major gluconeogenic substrate in ruminants [34,35]. *Prevotella*, the most dominant genus in the rumen, can ferment saccharides to succinate and acetate in the rumen [36]. Succinate is rapidly fermented to propionate by several ruminal microorganisms, via enzymatic processes involving coenzyme A [37]. *Saccharomyces cerevisiae* is capable of de novo biosynthesis of co-enzyme A, via the activities of dephospho-CoA kinase [38]. The results of our previous study [4] demonstrated that coenzyme A biosynthesis pathway was enriched in the rumen of beef steers fed diet supplemented with a *S. cerevisiae*-based additive. Thus, it is reasonable to infer that supplemental PROB and SYNB stimulated the conversion of succinate to propionate via biosynthesis of coenzyme A, which catalyzes the conversion of succinate to methylmalonyl-CoA and then to proponyl-CoA, which is subsequently converted to propionate [38].

The basal diet fed in this study contained over 9% dehydrated distillers grain (Table 1), which is known to contain high concentrations of polyunsaturated fatty acids [39]. Polyunsaturated fatty acids are very susceptible to degradation by oxygen species [40]. Up to 20 L of oxygen can enter the rumen of an adult ruminant daily, especially during meals, and as much as 3 mM of dissolved oxygen can be detected in rumen fluid [41]. 9-oxononanoic acid and several epoxyols, including 12,13-epoxy-9-hydroxy-10-octadecenoate and 9,10-epoxy-13-hydroxy-11-octadecenoate, are some of the major products of linoleic acid peroxidation [42]; while 8-iso-prostaglandin F 2α is often suggested to be an indicator of lipid peroxidation [43]. Thus, decreased concentrations of these aforementioned metabolites in steers fed supplemental PROB, compared to CON, are probably due to a reduced redox potential, possibly as a result of oxygen-scavenging activity of *S. cerevisiae*, one of the mechanisms of action by which its dietary supplementation improves rumen function [31,44,45]. In our previous study [4], supplementation of live yeast increased relative ruminal abundance of two microbial genes (ubiquinol-cytochrome c reductase cytochrome b subunit and cytochrome c oxidase subunit 2), which are components of the respiratory chain of *S. cerevisiae* that can catalyze the reduction of oxygen to water [46]. The fact that a similar result was not observed with supplemental SYNB, despite also containing *S. cerevisiae*, suggests that the lactic acid bacteria contained in supplemental PROB played a part. Lactic acid bacteria, including *E. lactis* and *L.* case, both of which are constituents of supplemental PROB, were demonstrated to have a high superoxide anion scavenging ability [47,48]. *Lactobacillus casei* was reported to reduce lipid peroxidation and improve lipid metabolism, both in blood and liver of rats [48]. *Lactobacillus paracasei* and other lactic acid bacteria were shown to prevent hydroxyl radical production [49]. Taken together, these results indicate that a reduced abundance of lipid peroxidation products caused by feeding supplemental PROB was either due to the antioxidant effects of lactic acid bacteria contained in supplemental PROB or a synergistic effect of both *S. cerevisiae* and lactic acid bacteria.

Amino acids are known to be susceptible to degradation by lipid oxidation products [50]. Histidine, and to a lesser extent, lysine are one of the most vulnerable amino acids to oxidative degradation [50,51]. Reduced concentrations of 2-amino-6-oxohexanoate and 6-acetamido-2-oxohexanoate, which are both intermediate products of lysine degradation [52], in steers fed supplemental PROB, are possibly caused by reduced ruminal concentrations of fatty acid oxidation products in these animals. This is supported by the results of our companion study, which showed a decreased ruminal ammonia-N concentration and increased ruminal concentrations of amino acids in steers fed supplemental PROB relative to CON [7]. Further studies are needed to determine how fatty acid oxidation products affect amino acid metabolism in the rumen.

## 5. Conclusions

This study demonstrated that dietary supplementation with either PROB or SYNB did not alter the functional capacity of the ruminal microbiome but altered the ruminal fermentation pattern towards increased propionate concentration. Supplemental PROB, but not SYNB, altered the ruminal carboxyl-metabolome towards reduced concentrations of metabolic products of fatty acid peroxidation and amino acid oxidative degradation. The significance of the carboxyl-metabolome alterations to ruminal fatty acid and amino acid metabolism and the influence on host performance should be explored in future studies.

## Figures and Tables

**Table 1:** Ingredient and chemical composition of the basal diet ^1^.

Ingredient (%DM)	% of Dietary DM
Corn silage	79.7
Dehydrated distillers grain	9.06
Soybean meal	9.28
Limestone	0.42
Deccox ^2^	0.03
Vitamin and mineral premix ^3^	1.51
Nutrient analysis ^4^	
DM, %	44.5
CP, %	14.7
aNDF, %	38.6
ADF, %	21.5
EE, %	3.50
Ca, %	0.87
P, %	0.63
TDN, %	72.6
NE_m_, Mcal/kg	1.72
NE_g_, Mcal/kg	1.10

^1^ Chemical composition of complete diets calculated from analysis and concentration of individual ingredients. ^2^ Contains 6% decoquinate for the prevention of coccidiosis (Zoetis Inc.). ^3^ Guaranteed analysis: 15% Ca; 7.5% P; 20% salt; 1% Mg; 1% K; 3600 mg/kg Mn; 12 mg/kg Co; 1200 mg/kg Cu; 3600 mg/kg Zn; 27 mg/kg Se; 60 mg/kg I; 660,000 IU/kg vitamin A; 660 IU/kg vitamin E; and 66,000 IU/kg vitamin D. ^4^ DM = dry matter; CP = crude protein; aNDF = neutral detergent fiber (amylase treated); ADF = acid detergent fiber; EE = ether extract; TDN = total digestible nutrients; NE_m_ = net energy of maintenance; and NE_g_ = net energy of gain. The chemical composition of the top-dressed premix was not included in the nutrient analysis.

**Table 2:** Identified ruminal metabolites that were altered in beef steers fed diets supplemented with direct-fed microbials containing multiple microbial species and their fermentation products.

Item	Normalized RT	FC	FDR
Effects of supplemental PROB			
2-amino-5-oxohexanoate	442.4	0.41	0.01
6-acetamido-2-oxohexanoate	638.7	0.45	0.01
9-oxononanoic acid	1256.5	0.69	0.02
Isomer of 9-oxononanoic acid *	1097.7	0.74	0.04
8-iso prostaglandin F1alpha	1245.5	0.82	0.01
9,10-epoxy-13-hydroxy-11-octadecenoate	1330.5	0.70	0.05
12,13-epoxy-9-hydroxy-10-octadecenoate	1337.4	0.70	0.05
Isomer of 12,13-epoxy-9-hydroxy-10-octadecenoate *	1364.4	0.72	0.05
Succinic acid	973.7	0.61	0.03
Hydroxylpropionic acid	424.9	1.52	0.01
Propionic acid	758.4	1.43	0.05
Isomer of propionic acid	723.7	1.71	0.01
Effects of supplemental SYNB			
Succinic acid	973.7	0.74	0.01
Pimelate	1374.2	0.54	0.03
Isomer of propionic acid	723.7	1.32	0.05
Propionic acid	758.4	1.54	0.02

Normalized RT (retention time) shows the corrected retention time of the peak pair with Universal RT Calibrant data. FC—fold change relative to Control; FDR—False discovery rate-adjusted *p-value*. * More than one isomer of the indicated metabolite was identified. PROB = a blend of *S. cerevisiae*, *Enterococcus lactis, Bacillus subtilis, Enterococcus faecium*, and *L. casei*, and their fermentation products fed at 19 g/steer/day (PMI, Arden Hills, MN); SYNB = a blend of live *S. cerevisiae* and the fermentation products of *S. cerevisiae, Enterococcus lactis*, *Bacillus licheniformis*, and *Bacillus subtilis* fed at 28 g/steer/day PMI, Arden Hills, MN). Only metabolites with both fold-change ≥ 1.2 or ≤ 0.83, relative to control and *p* ≤ 0.05 are shown.

## Data Availability

The data presented in this study are available on request from the corresponding author.

## Supplementary Materials

The following are available online at https://www.mdpi.com/2076-2615/11/1/72/s1. Figure S1: Linear discriminant analysis effect size comparisons of ruminal functional genes, Table S1: Relative abundance of microbial taxa, Table S2: List of peak pairs detected from CIL LC-MS measurement of the samples.
