# Supplementary material for: Effects of Multi-Species Direct-Fed Microbial Products on Ruminal Metatranscriptome and Carboxyl-Metabolome of Beef Steers
Source: Animals (Basel). 2021 Jan 2;11(1):72. doi: 10.3390/ani11010072 (PMC7823837; doi:10.3390/ani11010072)
Supplement: Supplementary file 1 [file animals-11-00072-s001.zip › Proof Metatranscriptome Supplementary Figure 1.docx]

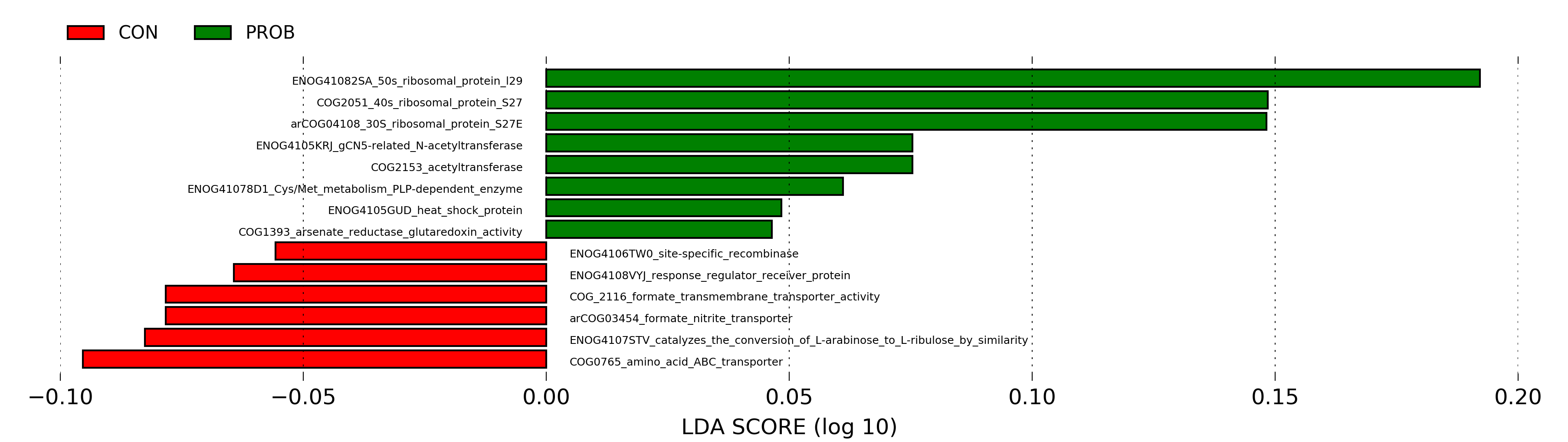


B


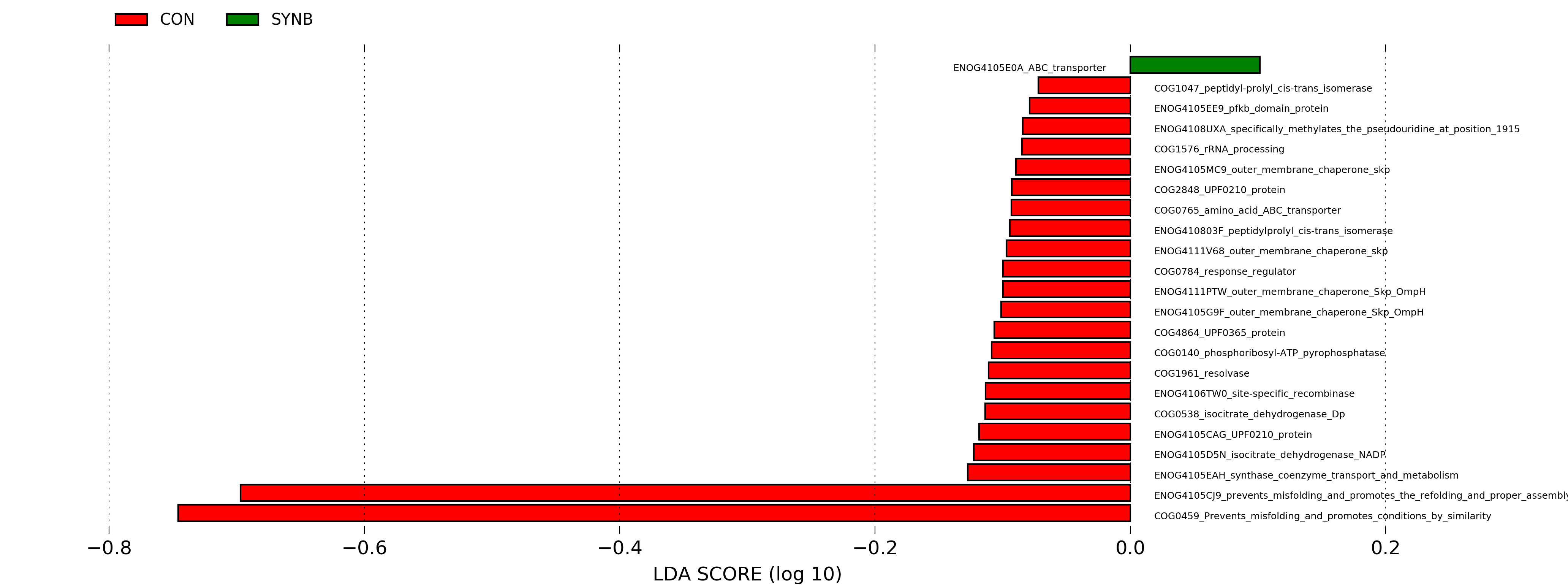


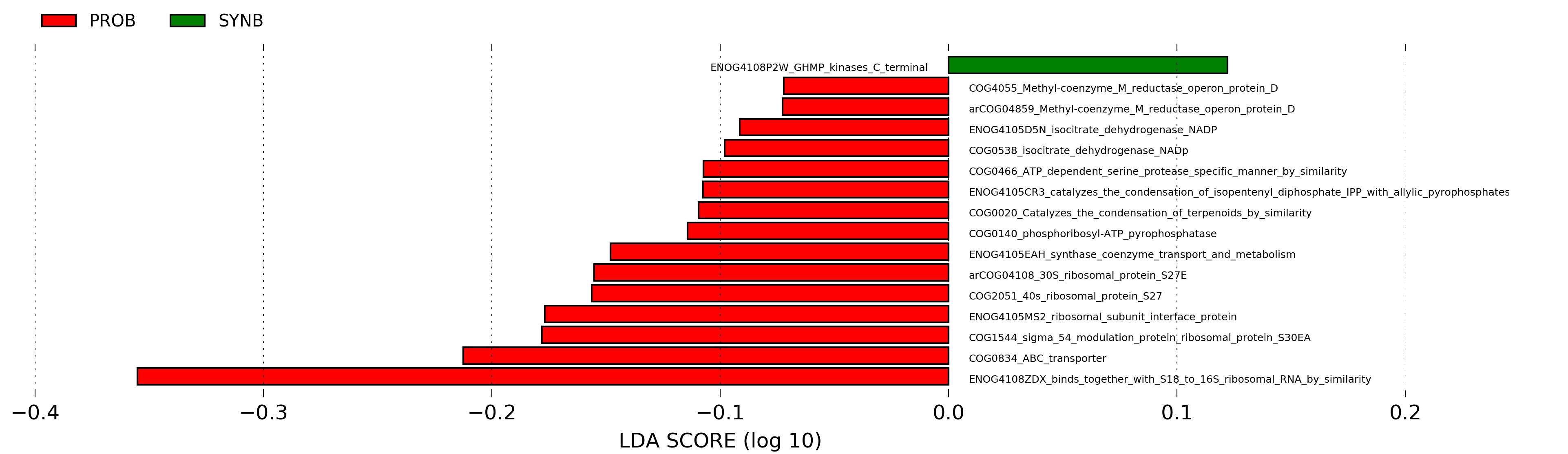


Supplementary Figure S1. **Linear discriminant analysis effect size** (LEfSe) comparisons of ruminal functional genes between CON vs. PROB (A), CON vs. SYNB (B), and PROB vs. SYNB (C).

CON = control; PROB = a blend of live *S. cerevisiae*, *Enterococcus lactis, Bacillus subtilis, Enterococcus faecium, and L. case*i, and their fermentation products fed at 19 g/steer/day (PMI, Arden Hills, MN); SYNB = a blend of live *S. cerevisiae* and the fermentation products of *S. cerevisiae, Enterococcus lactis*, *Bacillus licheniformis*, and *Bacillus subtilis* fed at 28 g/steer/day PMI, Arden Hills, MN)*.*
